# PD1-Targeted Transgene Delivery to Treg Cells

**DOI:** 10.3390/v16121940

**Published:** 2024-12-19

**Authors:** Vladislav A. Zhuchkov, Yulia E. Kravchenko, Elena I. Frolova, Stepan P. Chumakov

**Affiliations:** 1 Shemyakin-Ovchinnikov Institute of Bioorganic Chemistry, Russian Academy of Sciences, 117997 Moscow, Russia; 2 Biomarker Research Laboratory, Institute of Fundamental Medicine and Biology, Kazan Federal University, 420008 Kazan, Russia

**Keywords:** PD1, retargeting, lentivector, nanobody, Treg, FOXP3

## Abstract

Achieving the precise targeting of lentiviral vectors (LVs) to specific cell populations is crucial for effective gene therapy, particularly in cancer treatment where the modulation of the tumor microenvironment can enhance anti-tumor immunity. Programmed cell death protein 1 (PD-1) is overexpressed on activated tumor-infiltrating T lymphocytes, including regulatory T cells that suppress immune responses via FOXP3 expression. We developed PD1-targeted LVs by incorporating the anti-PD1 nanobody nb102c3 into receptor-blinded measles virus H and VSV-G_mut_ glycoproteins. We assessed the retargeting potential of nb102c3 and evaluated transduction efficiency in activated T lymphocytes. FOXP3 expression was suppressed using shRNA delivered by these LVs. Our results demonstrate that PD1-targeted LVs exerted pronounced tropism towards PD1^+^ cells, enabling the selective transduction of activated T lymphocytes while sparing naive T cells. The suppression of FOXP3 in Tregs reduced their suppressive activity. PD1-targeted glycoprotein H provided greater specificity, whereas the VSV-G_mut_, together with the anti-PD1 pseudoreceptor, achieved higher viral titers but was less selective. Our study demonstrates that PD1-targeted LVs may offer a novel strategy to modulate immune responses within the tumor microenvironment with the potential for developing new therapeutic strategies aimed at enhancing anti-tumor immunity.

## 1. Introduction

Existing gene therapy approaches are primarily designed to correct inherited genetic disorders and target malignant tumors [1]. A substantial proportion of these therapies rely on viral vectors for transgene delivery, including lentivirus-based vectors (LVs) [2]. Advantages of lentiviral vectors for gene therapy include high capacity and stable transgene expression due to integration of the expression cassette into the genome. LVs also exhibit low levels of silencing because of preferential integration into genomic regions where active gene expression occurs, a rare occurrence of neutralizing antibodies in the population [3], the ability to deliver the transgene to non-dividing cell populations [4], and the possibility of using different viral glycoproteins with varying tissue specificities [5]. The disadvantages include the risk of developing secondary pathologies due to disruption of important cellular genes from random integration [3], the possibility of mobilization of the integrated vector genome by endogenous retroviruses, and relatively limited targeted delivery of the transgene to specific cell populations [5]. Moreover, the in vivo use of the most common variant—the glycoprotein of vesicular stomatitis virus (VSV-G)—is ineffective due to the widespread expression of its receptor, low-density lipoprotein receptor (LDLR), on almost all cells of the body [6] and its susceptibility to inactivation by the serum complement system [7]. Integrase-deficient lentiviral vectors, which lack the ability to integrate the transgene cassette into the genome, can overcome the first two disadvantages—the risk of developing diseases due to integration into unfavorable genomic sites and the mobilization of the vector genome by endogenous retroviruses [8]. To address the third disadvantage, it is necessary to develop and test a variety of modified pseudotyping glycoproteins with altered tropism [9]. The use of modified glycoproteins with tropism toward various cellular receptors will enable the development of tools for conducting gene therapy in vivo, facilitating the delivery of transgenes to specific organs and tissues. This approach can expand the applications of lentiviral vectors, particularly by allowing targeted therapies for cancer or metabolic disorders.

LVs can be retargeted using modified glycoproteins from paramyxoviruses such as measles [10], Nipah [11], and Tupaia (TPMV) [12], as well as the Sindbis virus [13]. Recently, it has been demonstrated that a modified G protein of the vesicular stomatitis virus (VSV-G), combined with an auxiliary ScFv-displaying pseudoreceptor, can also be used for lentivector retargeting [14]. Targeting moieties include single-chain variable fragments (ScFv) [15], designed ankyrin repeat proteins (DARPins) [16], natural ligands of cellular receptors [17], and adapter molecules. These adapters enable retargeting by adding various soluble ligands that connect the adapter to the cellular receptor [18]. The most extensively studied and developed method for retargeting lentiviral vectors involves using a receptor-blinded H protein of the measles virus fused with a targeting molecule—a cytokine, DARPin, or ScFv [19]. A similar approach is applied when utilizing a receptor-blinded G protein of the Nipah virus [20]. The advantage of this pseudotyping method lies in its very high specificity of transduction, exclusively affecting cells that bear the target receptor on their surface. However, the drawbacks include a significantly reduced viral titer, typically at least an order of magnitude lower compared to lentiviral vectors pseudotyped with the intact H protein of the measles virus [21]. This method is incompatible with many known high-affinity ScFvs or DARPins; for effective cell infection via fusion of the viral and cellular membranes, the F glycoprotein must be at an optimal distance from the cell membrane and correctly oriented [22]. This requirement imposes constraints on the optimal length of the chain comprising the H protein, the targeting moiety, and the extracellular domain of the receptor [23]. Other methods for targeting that rely on a pseudoreceptor—an individual molecule anchored on the viral membrane, in combination with either the stalk domain of the measles virus H protein or a receptor-blinded G protein of VSV—have fewer limitations. However, employing a separate targeting receptor may be associated with reduced transduction specificity [24].

In addition to steric parameters, the interaction between the targeting domain within the H glycoprotein and the cellular receptor uniquely depends on the binding strength to the ligand: affinity values above a certain threshold do not influence the infectious titer, and this threshold inversely correlates with the abundance of target receptor on the cell membrane [25]. Collectively, this means that the selection of targeting molecules for retargeting lentiviruses must be conducted in a task-specific manner. From the perspective of size and ease of selection, nanobodies—the variable regions of camelid heavy-chain antibodies—are ideally suited to such applications due to their good solubility, compact size, and simplicity of selection from immune libraries [26]. Their small size reduces the overall bulkiness of the complex with the H protein, expanding the range of epitopes suitable for targeting within cellular receptors. Moreover, the straightforward process of nanobody selection [27] theoretically allows for the rapid generation of numerous nanobody variants against various epitopes, increasing the chances of identifying a variant that functions effectively within the H protein.

The ability to retarget lentiviral vectors to specific cellular receptors significantly enhances their therapeutic potential. In particular, applying these retargeted vectors to modulate immune cells within the tumor microenvironment offers a promising strategy for cancer therapy. Tumor progression is closely linked to interactions with immune system cells, which often leads to the establishment of a tumor microenvironment characterized by effector T lymphocytes with diminished cytotoxicity and an abundance of regulatory T lymphocytes (Tregs) that promote tumor tolerance by secreting immunosuppressive cytokines [28]. A notable feature of T lymphocytes within this microenvironment is the overexpression of the immune checkpoint PD1 (programmed cell death protein 1) [29,30]. Combined with the frequent overexpression of its ligand PD-L1 on tumor cells, this leads to senescence and exhaustion of intratumoral immune cells, thereby impairing anti-tumor immunity [31].

Current therapeutic strategies exploit this mechanism by using monoclonal antibodies to block PD1/PD-L1 signaling, aiming to reactivate exhausted T cells [32]. Moreover, the high expression of PD1 presents an opportunity to use it as a marker for delivery of genetic constructs into T lymphocytes via retargeted lentiviral vectors. By incorporating targeting moieties specific for PD1 into lentiviral vectors, it may be possible to selectively transduce T lymphocytes within the tumor microenvironment to modulate their functions or confer new properties.

Given that PD1 is overexpressed not only on effector but also on regulatory tumor-infiltrating T lymphocytes [33], delivering a construct that suppresses the function of Tregs could be a potential strategy to reduce immunological tolerance to the tumor. Regulatory T cells are characterized by expression of the transcription factor FOXP3, which directs the differentiation of naive CD4^+^ T lymphocytes toward a regulatory phenotype [34] and is essential for Tregs to maintain their function [35]. Suppressing FOXP3 through RNA interference leads to the loss of their regulatory phenotype [36]. Therefore, reducing the number of active Tregs within the tumor may shift the immunological balance from tolerance to an active anti-tumor response [37].

In this study, we developed several variants of PD-1-targeted lentiviral vectors to deliver shRNA against the transcription factor FOXP3, aiming to suppress regulatory T-cell activity. We compared these vectors in terms of their efficiency and specificity.

## 2. Materials and Methods

### 2.1. Plasmids and Cloning

The pLCMV-tagRFP, pLCMV-tagGFP and pLCMV-nanoLuc [38] lentiviral vector expression plasmids were constructed previously. PD1 lentiviral expression vector pLSF-PD1puro was constructed by inserting a full PDCD1 sequence PCR-amplified with PD1 Xba dir (AGAGATCTAGACTCCAGGCATGCAGATCCCAC) and PD1 Sal rev (AGAGAGGTCGACGGTCAGAGGGGCCAAGAG) and digested with corresponding endonucleases into pLSF-PL4-puro linearized by the same enzymes. The psPAX2 and pMD2.G were a gift from Didier Trono (Addgene plasmid # 12260 and 12259). The pCG-Hc∆18-AA was a gift from Jakob Reiser (Addgene plasmid # 86559). Intermediary plasmids pCG-Hc∆18-AAXB and pCG-4AHc∆24-AAXB were constructed from pCG-Hc∆18-AA via PCR amplification with the primers Hcd18 bsmbi dir (AGAGAGCGTCTCAGATCTCCCGGTAGTTAATTAAAAATTAGGG) or 4AHd24-BsmBi-gatc dir (AGAGAGCGTCTCAGATCTAGGGTGCAAGATCATCCACAATGGCCGCTGCAGCCAACCGGGAGCACCTGATG), respectively, and Hcd18-aa xba-bamh rev ATCCTCTTGCGGCCGCCGGATCCCTCTCTCTCTCTTCTCTTCTAGATTCCCGGGTGACTGTGC, followed by subsequent restriction of PCR products with BsmBI and NotI restriction endonucleases and cloning back into pCG-Hc∆18-AA digested with BamHI and NotI. This operation introduced unique XbaI and BamHI sites on the 3′-end of the H protein sequence, which were then used to clone the sequence of the 102c3 nanobody. The nb102c3 sequence was synthesized previously [39]. Measles F-protein- and H-protein-encoding plasmids pMD2-FΔ30 and pCG-4AHc∆24 were constructed previously [40]. A Nipah F-protein encoding plasmid pCG-NipF∆22 was constructed via the cloning of the truncated Nipah protein sequence bearing terminal BamHI and SpeI restriction sites synthesized de novo by Integrated DNA Technologies into pCG-Hc∆18-AA digested with BamHI and SpeI. The Nipah receptor-ablated G-protein-encoding plasmid pCG-Nip∆34Gm4-XB was constructed via the cloning of the truncated G-protein sequence (E501A, W504A, Q530A, E533A) synthesized de novo by Integrated DNA Technologies with the addition of the 5′-AGAGAGCGTCTCAGATCTAGGGTGCAAGATCATCCACA and TCTAGAAGAGAAGAGAGAGAGAGGGATCCGGCGGCCGCAAGAGGATCGCATCACCATCACCATCACTGATAGTTTGTACTAGTGTGAAATAGACATCAGTAA-3′ sequences digested with BglII and SpeI into pCG-Hc∆18-AA digested with BamHI and SpeI. pCG-Nip∆34Gm4-102c3 was subsequently made by cloning the nb102c3 sequence into pCG-Nip∆34Gm4-XB digested with XbaI and BamHI. A plasmid encoding receptor-ablated VSV-G protein pMD2.Gmut (I41L, K47Q, R354A) was constructed by cloning the DNA fragment made by three-piece overlap-extension PCR with the primers VSVG EcoRI dir (GCACGTGAGATCTGAATTCAACAGAGATCG) and VSVG 4147 rev (CCTTGTGACTCTGGGGCATTTTGACTTGTAAGGCTGTGCC), VSVG 4147 dir (GGCACAGCCTTACAAGTCAAAATGCCCCAGAGTCACAAGG) and VSVG 354 rev (CCAGTCATCCCACAGTTCGGCTTCTGTGGTAGTTCCACTG), and VSVG 354 dir (CAGTGGAACTACCACAGAAGCCGAACTGTGGGATGACTGG) and VSVG EcoRI rev (GCACTGGTGGGGTGAATTCC) performed on pMD2.G plasmid and digested with EcoRI back into pMD2.G digested with the same enzyme. The standalone pseudoreceptor plasmid pCG-VHHR was constructed by means of the cloning of a synthetic construct comprising Kozak and CD8 leader sequence (GCCACCATGGCCTTACCAGTGACCGCCTTGCTCCTGCCGCTGGCCTTGCTGCTCCACGCCGCCAGGCCG) followed by XbaI and BamHI recognition sites, a 12-amino-acid spacer sequence G-GAGAGCAAGTACGGACCACCATGTCCACCATGCCCG, and the CD28 transmembrane domain sequence followed by the NheI recognition site (TTTTGGGTGCTGGTGGTGGTTGGTGGAGTCCTGGCTTGCTATAGCTTGCTAGTAACAGTGGCCTTTATTATTTTCTGGGTGGCTAGCAGGAGTAAGAGG), which was extended on its 5′-terminus by means of PCR amplification with primers CD8s BglII dir (AGAGAGAGATCTGCCACCATGGCCTTACCAGTGACCG) and CD28TM NheI rev (CCTCTTACTCCTGCTAGCCACCC), and digested with BglII and NheI into pCG-Hc∆18-AA digested with BamHI and SpeI. pCG-102c3R was made by cloning the nb102c3 sequence into pCG-VHHR linearized with XbaI and BamHI. The shRNA-expressing lentivector pLH1-7SKP-shFOXP3 was constructed by cloning synthesized and annealed phosphorylated oligonucleotides shFOXP3-1 dir (GATCCGGCCACATTTCATGCACCAGCTGTGCTTGCTGGTGCATGAAATGTGGCTTTTTG) and shFOXP3-1 rev (AATTCAAAAAGCCACATTTCATGCACCAGCAAGCACAGCTGGTGCATGAAATGTGGCCG) into a pLH1p plasmid digested with BamHI and EcoRI to obtain pLH1p-shFOXP3-1, and then cloning shFOXP3-2 dir (GATCCGGAGTCTGCACAAGTGCTTTGTTGTGCTTACAAAGCACTTGTGCAGACTCTTTTTG) and shFOXP3-2 rev (AATTCAAAAAGAGTCTGCACAAGTGCTTTGTAAGCACAACAAAGCACTTGTGCAGACTCCG) into pL7SKP in a similar fashion to obtain pL7SKP-shFOXP3-2, followed by insertion of a 300 bp SalI-SpeI fragment from pL7SKP-shFOXP3-2 into pLH1p-shFOXP3-1 linearized with XhoI-SpeI. For negative control transductions, pLH1-shLUC expressing shRNA to firefly luciferase (CACTTACGCTGAGTACTTCGATGTGCTTCATCGAAGTACTCAGCGTAAG) was used, which was constructed previously.

### 2.2. Cells and Culturing

The HEK-293T embryonic kidney cell line and U937 AML cell line were purchased from ATCC (Manassas, VA, USA) and cultured in DMEM/F12 and RPMI1640 media (Paneco, Moscow, Russia), respectively. The culture medium contained 10% fetal bovine serum (FBS; Gibco, Waltham, MA, USA), penicillin–streptomycin, L-glutamine, and non-essential amino acids (Gibco). Human peripheral blood mononuclear cells (PBMCs) were isolated from the blood of healthy donors by means of gradient density centrifugation on a Ficoll 1.077 (Paneco) according to the standard protocol. CD4+ T lymphocytes were isolated with EasySep Human CD4+ T Cell Isolation Kit (STEMCELL Technologies, Vancouver, BC, Canada) according to the manufacturer’s protocol. PBMCs and T lymphocytes were cultured in an AIM-V medium (Thermo, Waltham, MA, USA). All cells were cultured under conditions of 37 °C and 5% CO_2_.

### 2.3. Lymphocyte Activation and iTreg Generation

For activation and PD1 induction, CD4+ T lymphocytes and PBMCs were cultured at a density of 1 × 10^6^ cells/mL in the presence of IL-2; IL-2 and PHA-M (phytohemagglutinin M-form); PMA (phorbol 12-myristate 13-acetate), ionomycin and PHA-M; or PMA, ionomycin, IL-2, IL-4, IL-21, IFNγ (interferon-γ) and PHA-M. IL-2, IL-4, IL-21 and IFNγ (Peprotech, Waltham, MA, USA) were supplemented at 20 ng/mL, PMA (Sigma, Burlington, MA, USA) was added at 50 ng/mL, ionomycin (Sigma) was added at 2 μM, and PHA-M (Gibco, USA) was used at a concentration of 1% *v*/*v*. Cells were cultured for 7 days with cytokine replenishment every third day. To generate iTregs, the medium was supplemented with 20 ng/mL IL-2, 5 ng/mL TGFβ (R&D Systems, Minneapolis, MN, USA), 10 nM all-trans retinoic acid, and 100 ng/mL rapamycin (both from Cayman Chemical, Ann Arbor, MI, USA) and cultured for 7 days with occasional cytokine replenishment.

### 2.4. Transfection and Lentivirus Packing

All of the transfections were performed with polyethyleneimine 25k (Polysciences, Warrington, PA, USA) according to the recommendations provided in [41]. The ratio of the plasmids for the syncytia-formation test was 5:7:1 (pLCMV-tagRFP:pMD2-FΔ30:pCG-4AHc∆24AA-102c3 or pCG-Hc∆18AA-102c3) or 4:5:1 (pLCMV-tagRFP:pCG-NipF∆22:pCG-Nip∆34Gm4-102c3), while for the lentivirus vector packaging, the ratios were 8:8:7:1 (lentivector:psPAX2:pMD2-F∆30:H-protein-coding plasmid), 6:6:5:1 (lentivector:psPAX2:pCG-NipF∆22:pCG-Nip∆34Gm4-102c3), and 5:3:2:3 (lentivector:psPAX2:pMD2.Gmut:pCG-102c3R). For lentivirus vector production, the day after transfection, the medium was changed to DMEM/F12 supplemented with PeproGrow serum replacement solution (Peprotech), and the virus-containing medium was later collected after 48 h of incubation, filtered through a 0.45 μm syringe filter, and used for the transduction of recipient cells via the standard spinoculation procedure with the addition of 8 μg/mL polybrene (Sigma) and 1 mg/mL Synperonic F-108 (Sigma). The cells transduced with fluorescent-protein-coding lentivector were examined 72 h post-transduction via a fluorescent microscope, and the viral titers were determined using flow cytometry. Unless otherwise mentioned, transductions with FP- and Luc-expressing lentiviral vectors were performed with MOI = 2, and transductions with shRNA-expressing vectors were performed with MOI = 5. For stable PD1-expressing cell line generation, transduced cells were cultured in standard medium supplemented with 1 ug/mL puromycin for 3 weeks. For stable tagGFP-expressing U937 cell line generation, cells were sorted 1 week after transduction on a FacsVantage SE cell sorter (Beckton-Dickinson, Franklin Lakes, NJ, USA). 

### 2.5. RNA Extraction and RT-qPCR

Total RNA was extracted from CD4+ T lymphocytes and PBMCs using ExtractRNA tri-reagent (Evrogen, Moscow, Russia) according to the manufacturer’s protocol. cDNA was synthesized using Magnus revertase (Evrogen) and oligo-dT primer at 53 °C for 40 min followed by revertase inactivation at 72 °C for 5 min. qPCR was carried out in 50 μL reaction mixtures on a Bio-Rad iCycler myIQ using HSTaq DNA polymerase (Evrogen) in three biological repetitions. For PDCD1 quantitation, a set of primers consisting of PD1 dir (CAGTTCCAAACCCTGGTGGT), PD1 rev (GGCTCCTATTGTCCCTCGTG), and PD1 probe (6FAM-TGCTGGGCAGCCTGGTGCTG-BHQ1) was used. FOXP3 was detected with a set of primers consisting of FOXP3 dir (CGGACCATCTTCTGGATGAG), FOXP3 rev (TTGTCGGATGATGCCACAG), and FOXP3 probe (6FAM-AGGCCCACCTGGCTGGGAAA-BHQ1). GAPDH was used as a reference transcript and was detected with GAPDH dir (GAAGGTGAAGGTCGGAGTC), GAPDH rev (GAAGATGGTGATGGGATTTC), and GAPDH probe (6FAM-CAAGCTTCCCGTTCTCAGCCT-BHQ1).

### 2.6. Luciferase Assay

A NanoLuc luminescence assay was performed with the NanoGlo luciferase assay kit (Promega, Madison, WI, USA) in white 96-well plates (SPL Life Sciences, Pocheon-si, Gyeonggi-do, Korea) according to the manufacturer’s protocol. Cell samples were normalized for viability with CelltiterGLO reagent (Promega) according to the manufacturer’s protocol. Luminescence readings were collected using a Triad microplate reader (Dynex Technologies, Chantilly, VA, USA).

### 2.7. TGFβ Detection

TGFβ levels in the iTreg cell culture medium were detected using Human TGF-beta 1 DuoSet (R&D Systems) in maxisorp 96-well plates (Nunc, Rochester, NY, USA) according to the manufacturer’s protocol. Cells were washed 3 times and seeded in an AIM-V medium with appropriate supplements excluding TGFβ. A control sample from each well was taken after seeding to serve as a reference. Cells were cultured for 48 h before measuring TGFβ levels. Absorbance readings were collected using a Triad microplate reader.

### 2.8. Statistical Analyses

The statistical analysis was performed using GraphPad Prism 10.

### 2.9. Manuscript Preparation

ChatGPT was utilized as a language refinement and editing tool during the manuscript preparation process. Specifically, it was employed to translate sections of the manuscript from Russian to English, ensuring scientific accuracy and readability, and to enhance wording, sentence structure, and overall flow to ensure the clarity and professional presentation of the content. All edits and suggestions provided by ChatGPT were reviewed and validated by the authors to ensure alignment with the scientific content and objectives of this manuscript.

## 3. Results

**Figure 1:**
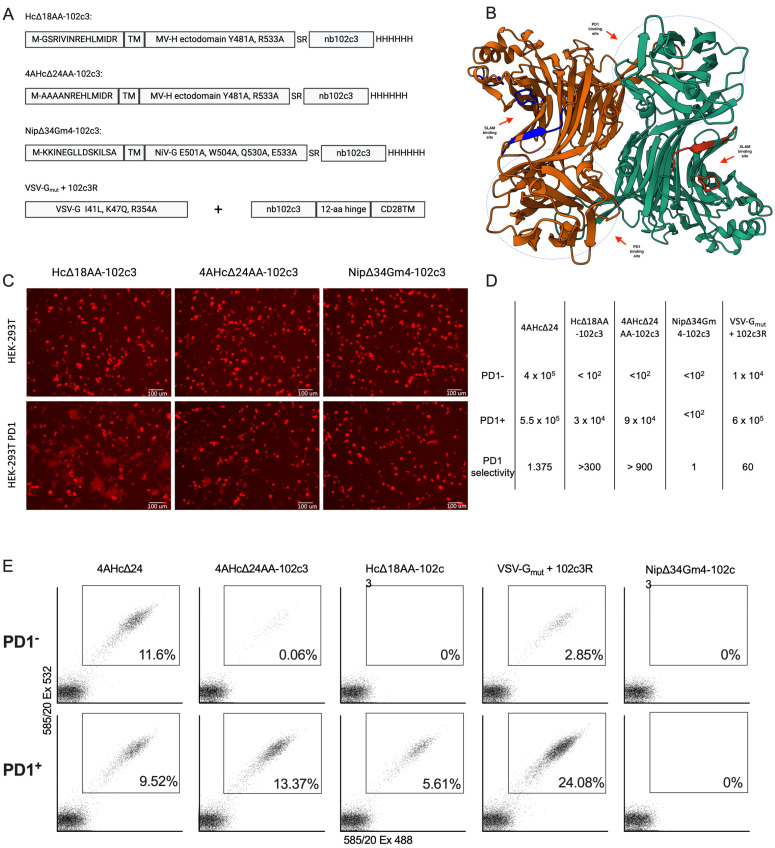
(**A**) Schematic representations of retargeted measles (Hc∆18AA-102c3, 4AHc∆24AA-102c3), Nipah (Nip∆34Gm4-102c3), and VSV (VSVG_mut_+102c3R) glycoproteins tested for lentivector retargeting. (**B**) Predicted structure of the homodimer of the measles H-protein head fused with nb102c3. (**C**) Microphotographs of the syncytia formation test performed on HEK-293T and HEK-293T-PD1 cells transfected with Hc∆18AA-102c3, 4AHc∆24AA-102c3, and Nip∆34Gm4-102c3 with corresponding F-protein-encoding plasmids and the lentivector plasmid encoding tagRFP (red channel). (**D**) Infectious titers of all retargeted LVs (i.u.) and selectivity (fold). (**E**) FACS plots of HEK-293T and HEK-293T-PD1 cells transduced with a corresponding lentivector carrying the tagRFP sequence (1 mL of non-concentrated lentivirus sample per 35 mm well).

To target lentiviral vectors to PD1-expressing cells, we used the nanobody nb102c3 [39]. Two receptor-blinded (Y481A, R533A) H proteins from the measles virus, with deletions of 18 and 24 N-terminal amino acids (Hc∆18AA-102c3 and 4AHc∆24AA-102c3), were designed, each fused at the C-terminus to the nb102c3 (Figure 1B). Additionally, a receptor-blinded G protein from the Nipah virus (E501A, W504A, Q530A, E533A) fused with nb102c3 was utilized. We also employed a receptor-blinded variant of the VSV-G protein (K47Q, R354A) combined with a membrane-anchored version of nb102c3 (Figure 1A).

For the initial evaluation of the ability of Hc∆18AA-102c3, 4AHc∆24AA-102c3, and Nip∆34Gm4-102c3 to mediate the receptor-dependent fusion of viral and cellular membranes, we transfected the HEK-293T and HEK-293T-PD1 cell lines with a plasmid mix carrying sequences for Hc∆18AA-102c3 or 4AHc∆24AA-102c3, F∆30, and the fluorescent marker tagRFP, or Nip∆34Gm4-102c3, NipF∆22, and tagRFP. Fluorescent syncytia formed in the HEK-293T-PD1 cells after transfection with Hd18AA-102c3 and 4AHc∆24AA-102c3, while no syncytia were observed in HEK-293T cells, indicating that the nb102c3 binding epitope within PD1 is suitable for retargeting lentiviral vectors pseudotyped with measles H protein to this receptor (Figure 1C). Transfection with Nip∆34Gm4-102c3 did not result in syncytia formation, suggesting that retargeting this glycoprotein with nb102c3 was ineffective. The VSVG_mut_+102c3R variant was not tested for syncytia formation because VSVG-mediated transduction typically results in few and small syncytia, making it less suitable for assessing the targeting molecule’s potential. The Hc∆18AA-102c3 construct produced the largest and most distinct syncytia.

Subsequently, four receptor-retargeted lentiviral vector variants, also carrying the tagRFP marker gene, were packaged and used to transduce the HEK-293T-PD1 and HEK-293T cell lines. Compared to the control vector packaged with intact 4AHc∆24, three variants showed increased tropism for HEK-293T-PD1 cells (Figure 1D,E and Figure S1). Packaging with Nip∆34Gm4-102c3 did not produce lentiviral particles with a detectable infectious titer. In contrast, retargeting using VSVG_mut_+102c3R resulted in a 5–7-fold higher transduction frequency of HEK-293T-PD1 cells compared to HEK-293T cells. However, neither Hc∆18AA-102c3 nor 4AHc∆24AA-102c3 effectively facilitated the transduction of HEK-293T cells. Only a few transduced cells were observed following transduction with 4AHc∆24AA-102c3 (Figure 1E). The VSVG_mut_+102c3R-pseudotyped variant achieved an approximately 8-fold higher infectious titer compared to 4AHc∆24AA-102c3, while the Hc∆18AA-102c3-pseudotyped variant had a titer about three times lower than that of 4AHc∆24AA-102c3.

**Figure 2:**
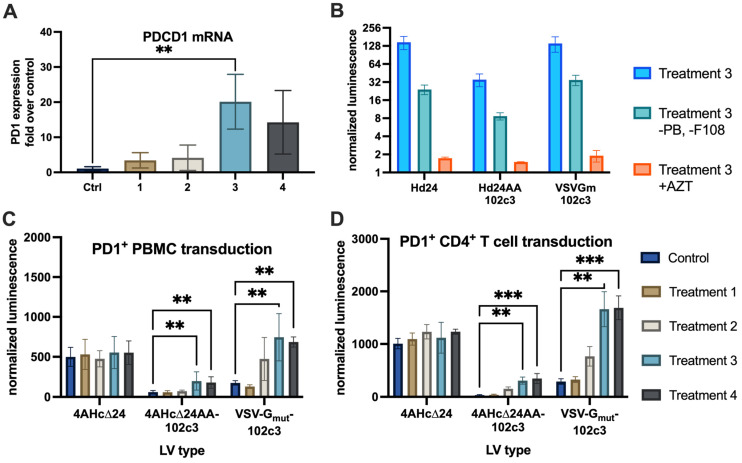
(**A**) qPCR quantitation of PDCD1 levels in PBMCs cultured with IL-2 (ctrl), IL-2 + PHA-M (1), PMA + ionomycin + PHA-M (2), IL-2 + PHA-M + PMA + ionomycin (3), and IL-2 + PHA-M + PMA + ionomycin + IFNγ + IL-4 + IL-12 (4), **—*p* = 0.007. (**B**) nanoLuc luminescence levels normalized to cell count after transduction with lentiviral vectors of CD4^+^ T cells with or without transduction enhancers (Treatment 3, as in (**A**), and Treatment 3-PB-F108), and in the presence of 50 µg/mL azidothymidine (Treatment 3 + AZT). (**C**) nanoLuc luminescence levels normalized to cell count after transduction with control (4AHc∆24) and targeted (4AHc∆24AA-102c3, VSVGmut+102c3R) lentivectors, **—*p* < 0.0075. (**D**) nanoLuc luminescence levels in transduced CD4^+^ T cells, **—*p* = 0.0018, ***—*p* = 0.0005.

To assess the transduction efficiency of retargeted lentiviral vectors in immune cells, it was necessary to first induce PD1 expression. We tested several cultivation conditions for peripheral blood mononuclear cells (PBMCs) and measured PDCD1 mRNA levels using qPCR (Figure 2A). The results indicated that cultivation with IL-2 + PHA-M + PMA + ionomycin resulted in the highest induction of PD1 expression.

Next, we used three variants of lentiviral vectors pseudotyped with 4AHc∆24AA-102c3, VSVG_mut_+102c3R, and 4AHc∆24 to deliver an expression cassette containing the nanoLuc (nLuc) luciferase gene into PD1-induced PBMCs (Figure 2C) and PD1-induced CD4^+^ T lymphocytes (Figure 2D). Analysis of luminescent activity revealed a correlation between PDCD1 expression levels and transduction efficiency for the 4AHc∆24AA-102c3- and VSVG_mut_+102c3R-pseudotyped lentivectors, a relationship that was not observed for the 4AHc∆24-pseudotyped vector. A significant difference in luminescence levels was observed between the control cells and those cultured in the presence of IL-2 + PHA-M + PMA + ionomycin, or IL-2 + PHA-M + PMA + ionomycin combined with IFNγ, IL-4, and IL-12, in samples transduced with vectors pseudotyped with 4AHc∆24AA-102c3 and VSVG_mut_+102c3R. Notably, the cells transduced with LVs pseudotyped with VSVG_mut_+102c3R showed higher levels of nLuc activity compared to cells transduced with 4AHc∆24AA-102c3-pseudotyped LVs, consistent with the data obtained from the HEK-293T-PD1 and HEK-293T cell line experiments. To assess the contribution of transduction enhancers and pseudotransduction to the observed effect, we transduced PD1^+^ CD4^+^ T lymphocytes in the absence of polybrene and Synperonic F-108 and after treatment with azidothymidine (Figure 2B). The absence of enhancers reduced transduction efficiency by approximately fivefold, while treatment with azidothymidine decreased luminescence intensity to near-background levels.

**Figure 3:**
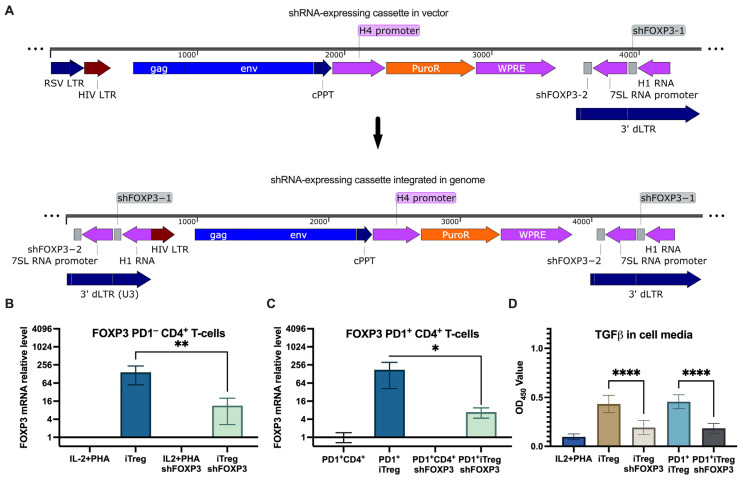
shRNA knockdown of FOXP3 with a non-targeted lentivector. (**A**) Schematic representation of shRNA-expressing lentivector and its cassette integrated in the genome. (**B**) qPCR quantitation of FOXP3 expression in stimulated CD4^+^ T cells and Tregs transduced with shFOXP3 and control shRNA, **—*p* = 0.0065. (**C**) qPCR quantitation of FOXP3 expression in PD1^+^ CD4^+^ T cells and PD1^+^ Tregs transduced with shFOXP3 and control shRNA, *—*p* = 0.015. (**D**) TGFβ levels in media of PD1^−^ and PD1^+^ Tregs transduced with shFOXP3 and the control, ****—*p* < 0.0001.

To suppress FOXP3 expression, we used a lentiviral construct designed for the simultaneous expression of multiple shRNAs [42] (Figure 3A). This construct, expressing two shRNAs targeting FOXP3, was tested in a lentiviral vector pseudotyped with 4AHc∆24 on induced Tregs (iTregs) derived from peripheral CD4^+^ T lymphocytes. Transduction of iTregs resulted in a significant reduction in FOXP3 mRNA levels, as measured by qPCR (Figure 3B).

Next, we investigated whether the induction of PD1 in iTregs affects the efficiency of transduction with the shFOXP3-expressing lentiviral vector pseudotyped with 4AHc∆24 (Figure 3C). The results showed that the induction of PD1 had only a minor impact on the transduction efficiency, as indicated by the decrease in FOXP3 mRNA levels.

To evaluate how FOXP3 suppression affects the immunosuppressive capacity of Tregs, we measured TGFβ levels in the cell culture media. The results (Figure 3D) demonstrated that shFOXP3-treated iTregs significantly reduced TGFβ production, indicating functional suppression of the regulatory phenotype. Notably, TGFβ production levels were similar between iTregs with high and low levels of PD1 expression.

**Figure 4:**
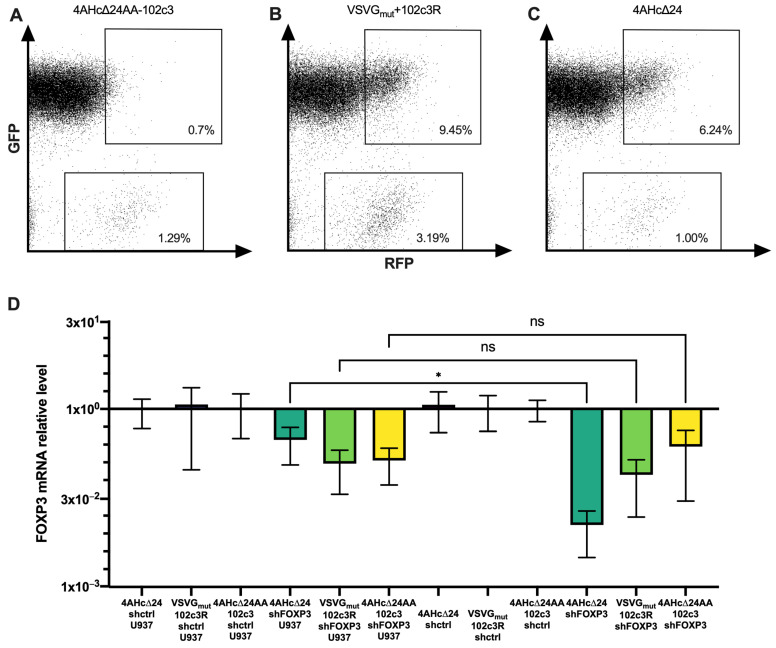
Cytometry measurements of CD4^+^ PD1^+^ T lymphocytes co-cultured with U937-tagGFP and transduced with 4AHc∆24AA-102c3-pseudotyped (**A**) VSVG_mut_+102c3R-pseudotyped (**B**) and 4AHc∆24-pseudotyped (**C**) LVs carrying the tagRFP sequence. (**D**) FOXP3 expression in PD1^+^ Tregs co-cultured with U937 cells and transduced with different pseudotypes of LVs carrying shFOXP3 or control shRNA. *—*p* = 0.0485, ns—*p* > 0.05.

To evaluate the selectivity of PD1-targeted lentiviral vectors in delivering transgenes to PD1^+^ cells, we used lentiviral particles carrying the tagRFP marker protein sequence, pseudotyped with 4AHc∆24AA-102c3, VSVG_mut_+102c3R, and 4AHc∆24. These were used to transduce CD4^+^ PD1^+^ T lymphocytes during co-cultivation with U937 cells expressing the tagGFP marker at a 1:10 ratio. As shown in Figure 4C, the non-targeted lentivector packaged with 4AHc∆24 efficiently transduced the “buffer” U937-tagGFP cells, while the proportion of fluorescent CD4^+^ PD1^+^ T lymphocytes reached 144% of that of U937-tagGFP cells. Transduction with the VSVG_mut_+102c3R-pseudotyped lentivirus increased the proportion of transduced CD4^+^ PD1^+^ T lymphocytes up to 303% (Figure 4B). Similarly, using lentiviral particles pseudotyped with 4AHc∆24AA-102c3 resulted in the exclusive transduction of T lymphocytes, with no distinct transduced population observed in buffer cells, and the proportion of T cells to U937-tagGFP falling within tagRFP^+^ gates rose to at least 1675% (Figure 4A and Figure S2).

Next, we examined how effectively FOXP3 was suppressed upon transduction of PD1^+^ iTregs co-cultured with U937 cells. As shown in Figure 4D, FOXP3 suppression occurred with all packaging constructs used in conjunction with the shFOXP3 vector. However, when comparing iTregs transduced alone to those co-cultured with U937, a significant difference in FOXP3 transcript levels was observed only for lentivectors pseudotyped with 4AHc∆24. In contrast, for LVs with shFOXP3 pseudotyped with VSVG_mut_+102c3R and 4AHc∆24AA-102c3, there were no significant differences in FOXP3 levels between transduced Tregs cultured alone and those co-cultured with U937. This indicates that the presence of PD1^−^ cells does not significantly affect FOXP3 suppression by PD1-targeted lentivectors.

## 4. Discussion

In this study, we developed PD1-targeted lentiviral vectors using nb102c3 and demonstrated their potential to selectively transduce activated T lymphocytes. We showed that suppressing FOXP3 expression in regulatory T cells (Tregs) using these vectors can reduce Treg activity, potentially enhancing anti-tumor immune responses.

Achieving the precise targeting of lentiviral vectors to specific cell populations remains a key obstacle to the direct in vivo application of lentiviral gene therapy. VSV-G pseudotyping, which is the most commonly used method for conferring broad tropism, is unsuitable for systemic application due to strong antiviral immune responses to the lentivector [43]. Additionally, the ubiquitous expression of its receptor (LDLR) results in only a small fraction of lentiviral vectors reaching the intended target tissue or organ [44]. Instead, systemically administered lentiviral vectors predominantly accumulate in the liver and spleen [45]. In cancer therapy, direct intratumoral injection can enhance the targeting of lentiviral vectors [46]; however, this approach still results in the simultaneous transduction of both tumor cells and cells within the tumor microenvironment [47], which is undesirable for many therapeutic strategies. The most extensively developed method for targeting lentiviral vectors involves using a receptor-blinded glycoprotein from the measles virus. This approach has been employed in studies targeting tumor cells via tumor-associated antigens such as HER2 [48], EGFR [10], and EpCAM [49] or immune cells through immunological receptors like CD20 [15], CD19 [50], CD8 [51], CD4 [52], and CD30 [23]. A receptor-blinded variant of VSV-G was described relatively recently [14], following the resolution of the crystal structure of the VSV-G and LDLR complex [53]. This advancement led to studies on retargeting lentiviral vectors using mutant VSV-G and a targeting pseudoreceptor co-expressed on packaging cells [24,54]. A distinctive limitation of this approach—previously applied to other viral glycoproteins [21,55]—is its incomplete selectivity of transduction. Since VSV-G_mut_ retains its fusogenic activity and is not physically linked to the targeting pseudoreceptor, spontaneous transduction of non-target cells can occur [14]. Nevertheless, lentiviral vectors pseudotyped with VSV-G_mut_ exhibit significantly higher infectious titers compared to those pseudotyped with glycoproteins from the measles virus and other paramyxoviruses. This higher titer can make the use of VSV-G_mut_ economically attractive for applications where preferential tropism, rather than absolute specificity, is sufficient.

Targeting lentiviral vectors at T lymphocyte receptors could enable the direct generation of CAR-T cells within the body [56], potentially reducing the cost of such therapies and offering new ways to modulate immune system activity. However, therapeutic approaches specifically aimed at the tumor microenvironment [47] require lymphocyte markers that are more abundantly expressed on immune cells within tumors than in the rest of the body. To overcome these challenges, targeting lentiviral vectors at specific receptors such as PD1 presents a promising strategy. PD1 is overexpressed on activated tumor-infiltrating T lymphocytes and is already a target for anti-cancer agents like checkpoint inhibitors [57]. Its elevated expression in the tumor microenvironment makes it an ideal candidate for directing lentiviral vectors to modulate immune responses [58].

We initially assessed nb102c3 as a retargeting moiety using a syncytium formation assay [10]. This assay allowed us to determine the ability of the modified paramyxovirus glycoproteins to mediate cell–cell fusion in PD1-expressing cells. The assay allowed us to bypass the need to produce pseudotyped lentiviral vectors and perform secondary transduction during the initial screening of candidate molecules. However, while the formation of syncytia is a necessary condition, it is not sufficient to confirm that a given targeting molecule can be used for lentiviral vector pseudotyping [59]. In our experiments, nb102c3 induced syncytia formation in HEK-293T-PD1 cells when fused to the measles virus H protein but not when fused to the Nipah virus G protein. Additionally, comparing two truncated variants of the measles virus H protein as carriers for the targeting molecule nb102c3, we found that the Hc∆18 variant was less effective, as evidenced by a lower infectious titer. This finding is consistent with earlier studies where the use of the 4AHc∆24 variant was preferable [60]. We utilized a receptor-blinded variant of the H protein with Y481A and R533A substitutions and observed no syncytia formation when transfecting PD1-negative cells. However, during transduction experiments with HEK-293-PD1 and HEK-293 cells, a minor population of transduced PD1-negative cells was detected, with a selectivity index of approximately 900×. This could be attributed to the presence of an additional, unblocked CD46 binding site within the H protein [61]. It is plausible that introducing further substitutions could enhance the selectivity index even further.

Next, we evaluated the efficiency of transduction in activated T lymphocytes to confirm the potential of our targeted vectors in a more physiologically relevant context. Our results demonstrated a clear correlation between PD1 expression levels and transduction efficiency in activated T lymphocytes, confirming the specificity of our PD1-targeted vectors. Importantly, naive T cells appeared not to be susceptible to transduction, potentially making transgene delivery specific to sites of inflammation, such as the tumor microenvironment.

Gene therapy targeting PD1-positive intratumoral T lymphocytes can aim to either enhance the cytotoxicity of effector cells or to suppress regulatory T lymphocytes (Tregs). Targeting FOXP3 in PD1-positive Tregs offers a strategy to reduce immunological tolerance within tumors. Suppressing FOXP3 disrupts Treg function without affecting effector T cells because this transcription factor is essential for maintaining the regulatory phenotype of T cells and is not involved in the function of effector T cells. Existing research indicates that the shRNA-mediated knockdown of FOXP3 leads to reduced activity of Tregs [36,62], which is corroborated by our experimental results. Furthermore, targeting Tregs is justified because existing therapeutic PD1-directed checkpoint inhibitors enhance effector T-cell activity by blocking the suppressive PD-L1/PD-1 signaling pathway [63], which may inadvertently stimulate Treg function, potentially causing disease hyperprogression in response to therapy [64]. Suppressing FOXP3 in intratumoral Tregs using retargeted lentiviral vectors could address this issue. Alternatively, LVs targeted at PD1 may be used to knock down PD1 expression itself, which could enhance the anti-tumor immune response [65]. Since nb102c3 can block signaling through the PD1/PD-L1 axis, it is plausible that lentiviral vectors targeted with this nanobody might independently suppress PD1-dependent T-cell suppression. However, achieving a noticeable effect would likely require extremely high multiplicities of infection (MOI).

We employed two types of retargeted glycoproteins to deliver the transgene into PD1-positive cells. Our experiments demonstrated that these glycoproteins differ in both the infectious titer achieved and their selectivity. Specifically, the combination of 4AHc∆24AA-102c3 with F∆30 resulted in the most selective transduction of PD1^+^ cells. However, the infectious titer of these retargeted lentiviral vectors was significantly lower than that obtained using the combination of VSV-G_mut_ with 102c3R, which, in contrast, exhibited substantially less selectivity toward PD1^+^ cells. These findings suggest that in in vivo applications, the reduced selectivity of VSV-G_mut_ could lead to more pronounced off-target effects.

Regulatory T lymphocytes play a crucial role in maintaining immune homeostasis. However, the excessive suppression of FOXP3—whether due to lentiviral vectors with low transduction specificity or highly specific vectors administered at excessively high doses—could potentially result in autoimmune disorders and hyperinflammation. These risks highlight important limitations that may constrain the therapeutic application of this approach. Another limitation is the cross-species variability of PD1, hindering the testing of our vectors in animal models [66]. Future work could involve engineering nanobodies that recognize murine PD1 or using humanized mouse models. Furthermore, it is challenging to thoroughly assess how the targeted delivery of shRNA against FOXP3 stimulates lymphocyte cytotoxic activity in co-cultures with tumor cells compared to non-targeted delivery. This difficulty arises because, in such experiments, tumor (PD-1^−^) cells are used in smaller proportions relative to immune (PD-1^+^) cells. Consequently, off-target transduction would be insignificant even when using non-retargeted LVs, making it difficult to evaluate differences in transduction efficiency between targeted and non-targeted LVs.

Beyond targeting tumors, suppressing FOXP3 in PD1^+^ T cells may also be useful in addressing immunological anergy associated with bacterial infections. For instance, evidence suggests that a similar immune response is observed in the inflammatory lesions caused by *Mycobacterium leprae* infection [67]. Furthermore, as PD1 expression has been observed on tumor cells themselves [68], our vectors could be adapted for direct anti-tumor strategies.

Overall, our study demonstrates the feasibility of using the nanobody nb102c3 to retarget lentiviral vectors to PD1-positive T lymphocytes, providing a novel tool for modulating immune responses within the tumor microenvironment. By selectively suppressing FOXP3 in Tregs, we offer a potential strategy to enhance anti-tumor immunity.

## 5. Conclusions

1. The anti-PD1 nb102c3 can effectively retarget lentiviral vectors toward PD1^+^ cell populations, both when incorporated into receptor-blinded measles virus glycoprotein and in combination with VSV-G_mut_. The former option provides greater specificity, while the latter achieves higher viral titers.
2. The efficiency of transduction depends on the levels of PD1 on immune cells, with older cells which express more PD1 transducing more effectively.
3. FOXP3 knockdown using dual shRNA significantly reduces the suppressive activity of regulatory T cells in vitro.
4. In the setting where a high number of PD1^−^ cells are present, the use of PD1-targeted lentiviral vectors significantly enhances Treg suppression compared to nontargeted vectors. This approach could be employed to develop new therapeutic strategies aimed at reprogramming the tumor microenvironment to promote a stronger anti-tumor response.

## Data Availability

The data presented in this study are available in this manuscript.

## Supplementary Materials

The following supporting information can be downloaded at: https://www.mdpi.com/article/10.3390/v16121940/s1, Figure S1: Control cytomentry measurements and targeting construct expression levels; Figure S2: Cytometry analysis of PD1^+^ CD4^+^ T lymphocytes transduced with tagRFP-expressing pseudotyped lentivectors.

## References

[1] Li X., Le Y., Zhang Z., Nian X., Liu B., Yang X. (2023). Viral Vector-Based Gene Therapy. Int. J. Mol. Sci..

[2] Milone M.C., O’Doherty U. (2018). Clinical use of lentiviral vectors. Leukemia.

[3] Wu C., Dunbar C.E. (2011). Stem cell gene therapy: The risks of insertional mutagenesis and approaches to minimize genotoxicity. Front. Med..

[4] Miyoshi H., Blomer U., Takahashi M., Gage F.H., Verma I.M. (1998). Development of a self-inactivating lentivirus vector. J. Virol..

[5] Joglekar A.V., Sandoval S. (2017). Pseudotyped Lentiviral Vectors: One Vector, Many Guises. Hum. Gene Ther. Methods.

[6] Burns J.C., Friedmann T., Driever W., Burrascano M., Yee J.K. (1993). Vesicular stomatitis virus G glycoprotein pseudotyped retroviral vectors: Concentration to very high titer and efficient gene transfer into mammalian and nonmammalian cells. Proc. Natl. Acad. Sci. USA.

[7] DePolo N.J., Reed J.D., Sheridan P.L., Townsend K., Sauter S.L., Jolly D.J., Dubensky T.W. (2000). VSV-G pseudotyped lentiviral vector particles produced in human cells are inactivated by human serum. Mol. Ther..

[8] Gurumoorthy N., Nordin F., Tye G.J., Wan Kamarul Zaman W.S., Ng M.H. (2022). Non-Integrating Lentiviral Vectors in Clinical Applications: A Glance Through. Biomedicines.

[9] Frank A.M., Buchholz C.J. (2019). Surface-Engineered Lentiviral Vectors for Selective Gene Transfer into Subtypes of Lymphocytes. Mol. Ther. Methods Clin. Dev..

[10] Nakamura T., Peng K.W., Vongpunsawad S., Harvey M., Mizuguchi H., Hayakawa T., Cattaneo R., Russell S.J. (2004). Antibody-targeted cell fusion. Nat. Biotechnol..

[11] Palomares K., Vigant F., Van Handel B., Pernet O., Chikere K., Hong P., Sherman S.P., Patterson M., An D.S., Lowry W.E. (2013). Nipah virus envelope-pseudotyped lentiviruses efficiently target ephrinB2-positive stem cell populations in vitro and bypass the liver sink when administered in vivo. J. Virol..

[12] Enkirch T., Kneissl S., Hoyler B., Ungerechts G., Stremmel W., Buchholz C.J., Springfeld C. (2013). Targeted lentiviral vectors pseudotyped with the Tupaia paramyxovirus glycoproteins. Gene Ther..

[13] Morizono K., Bristol G., Xie Y.M., Kung S.K., Chen I.S. (2001). Antibody-directed targeting of retroviral vectors via cell surface antigens. J. Virol..

[14] Dobson C.S., Reich A.N., Gaglione S., Smith B.E., Kim E.J., Dong J., Ronsard L., Okonkwo V., Lingwood D., Dougan M. (2022). Antigen identification and high-throughput interaction mapping by reprogramming viral entry. Nat. Methods.

[15] Funke S., Maisner A., Muhlebach M.D., Koehl U., Grez M., Cattaneo R., Cichutek K., Buchholz C.J. (2008). Targeted cell entry of lentiviral vectors. Mol. Ther..

[16] Munch R.C., Muhlebach M.D., Schaser T., Kneissl S., Jost C., Pluckthun A., Cichutek K., Buchholz C.J. (2011). DARPins: An efficient targeting domain for lentiviral vectors. Mol. Ther..

[17] Ou W., Marino M.P., Suzuki A., Joshi B., Husain S.R., Maisner A., Galanis E., Puri R.K., Reiser J. (2012). Specific targeting of human interleukin (IL)-13 receptor alpha2-positive cells with lentiviral vectors displaying IL-13. Hum. Gene Ther. Methods.

[18] Cordes N., Winter N., Kolbe C., Kotter B., Mittelstaet J., Assenmacher M., Cathomen T., Kaiser A., Schaser T. (2022). Adapter-Mediated Transduction with Lentiviral Vectors: A Novel Tool for Cell-Type-Specific Gene Transfer. Viruses.

[19] Levy C., Verhoeyen E., Cosset F.L. (2015). Surface engineering of lentiviral vectors for gene transfer into gene therapy target cells. Curr. Opin. Pharmacol..

[20] Bender R.R., Muth A., Schneider I.C., Friedel T., Hartmann J., Pluckthun A., Maisner A., Buchholz C.J. (2016). Receptor-Targeted Nipah Virus Glycoproteins Improve Cell-Type Selective Gene Delivery and Reveal a Preference for Membrane-Proximal Cell Attachment. PLoS Pathog..

[21] Rasbach A., Abel T., Munch R.C., Boller K., Schneider-Schaulies J., Buchholz C.J. (2013). The receptor attachment function of measles virus hemagglutinin can be replaced with an autonomous protein that binds Her2/neu while maintaining its fusion-helper function. J. Virol..

[22] Aguilar H.C., Henderson B.A., Zamora J.L., Johnston G.P. (2016). Paramyxovirus Glycoproteins and the Membrane Fusion Process. Curr. Clin. Microbiol. Rep..

[23] Friedel T., Hanisch L.J., Muth A., Honegger A., Abken H., Pluckthun A., Buchholz C.J., Schneider I.C. (2015). Receptor-targeted lentiviral vectors are exceptionally sensitive toward the biophysical properties of the displayed single-chain Fv. Protein Eng. Des. Sel..

[24] Strebinger D., Frangieh C.J., Friedrich M.J., Faure G., Macrae R.K., Zhang F. (2023). Cell type-specific delivery by modular envelope design. Nat. Commun..

[25] Hasegawa K., Hu C., Nakamura T., Marks J.D., Russell S.J., Peng K.W. (2007). Affinity thresholds for membrane fusion triggering by viral glycoproteins. J. Virol..

[26] Muyldermans S. (2013). Nanobodies: Natural single-domain antibodies. Annu. Rev. Biochem..

[27] Maass D.R., Sepulveda J., Pernthaner A., Shoemaker C.B. (2007). Alpaca (Lama pacos) as a convenient source of recombinant camelid heavy chain antibodies (VHHs). J. Immunol. Methods.

[28] Salemme V., Centonze G., Cavallo F., Defilippi P., Conti L. (2021). The Crosstalk between Tumor Cells and the Immune Microenvironment in Breast Cancer: Implications for Immunotherapy. Front. Oncol..

[29] Bally A.P., Austin J.W., Boss J.M. (2016). Genetic and Epigenetic Regulation of PD-1 Expression. J. Immunol..

[30] Austin J.W., Lu P., Majumder P., Ahmed R., Boss J.M. (2014). STAT3, STAT4, NFATc1, and CTCF regulate PD-1 through multiple novel regulatory regions in murine T cells. J. Immunol..

[31] Mao X., Xu J., Wang W., Liang C., Hua J., Liu J., Zhang B., Meng Q., Yu X., Shi S. (2021). Crosstalk between cancer-associated fibroblasts and immune cells in the tumor microenvironment: New findings and future perspectives. Mol. Cancer.

[32] Stark M.C., Joubert A.M., Visagie M.H. (2023). Molecular Farming of Pembrolizumab and Nivolumab. Int. J. Mol. Sci..

[33] Tanaka A., Sakaguchi S. (2017). Regulatory T cells in cancer immunotherapy. Cell Res..

[34] Fontenot J.D., Gavin M.A., Rudensky A.Y. (2003). Foxp3 programs the development and function of CD4^+^CD25^+^ regulatory T cells. Nat. Immunol..

[35] Wan Y.Y., Flavell R.A. (2007). Regulatory T-cell functions are subverted and converted owing to attenuated Foxp3 expression. Nature.

[36] Sun L., Yi S.N., Chen L. (2011). Effects of Foxp3 knockdown on the functions of human regulatory T cells. Zhonghua Yi Xue Za Zhi.

[37] Colbeck E.J., Jones E., Hindley J.P., Smart K., Schulz R., Browne M., Cutting S., Williams A., Parry L., Godkin A. (2017). Treg Depletion Licenses T Cell-Driven HEV Neogenesis and Promotes Tumor Destruction. Cancer Immunol. Res..

[38] Shramova E.I., Chumakov S.P., Shipunova V.O., Ryabova A.V., Telegin G.B., Kabashin A.V., Deyev S.M., Proshkina G.M. (2022). Genetically encoded BRET-activated photodynamic therapy for the treatment of deep-seated tumors. Light. Sci. Appl..

[39] Kalinin R.S., Ukrainskaya V.M., Chumakov S.P., Moysenovich A.M., Tereshchuk V.M., Volkov D.V., Pershin D.S., Maksimov E.G., Zhang H., Maschan M.A. (2021). Engineered Removal of PD-1 From the Surface of CD19 CAR-T Cells Results in Increased Activation and Diminished Survival. Front. Mol. Biosci..

[40] Kravchenko Y., Gagarinskaya D., Frolova E., Chumakov S. (2018). Chimeric antigen receptor expression in natural killer cell line NK-92 by transduction with lentiviral particles pseudotyped with the surface glycoproteins of the measles virus vaccine strain. Bull. Russ. State Med. Univ..

[41] Fukumoto Y., Obata Y., Ishibashi K., Tamura N., Kikuchi I., Aoyama K., Hattori Y., Tsuda K., Nakayama Y., Yamaguchi N. (2010). Cost-effective gene transfection by DNA compaction at pH 4.0 using acidified, long shelf-life polyethylenimine. Cytotechnology.

[42] Chumakov S.P., Kravchenko J.E., Prassolov V.S., Frolova E.I., Chumakov P.M. (2010). Efficient downregulation of multiple mRNA targets with a single shRNA-expressing lentiviral vector. Plasmid.

[43] Pichlmair A., Diebold S.S., Gschmeissner S., Takeuchi Y., Ikeda Y., Collins M.K., Reis e Sousa C. (2007). Tubulovesicular structures within vesicular stomatitis virus G protein-pseudotyped lentiviral vector preparations carry DNA and stimulate antiviral responses via Toll-like receptor 9. J. Virol..

[44] Duverge A., Negroni M. (2020). Pseudotyping Lentiviral Vectors: When the Clothes Make the Virus. Viruses.

[45] Pan D., Gunther R., Duan W., Wendell S., Kaemmerer W., Kafri T., Verma I.M., Whitley C.B. (2002). Biodistribution and toxicity studies of VSVG-pseudotyped lentiviral vector after intravenous administration in mice with the observation of in vivo transduction of bone marrow. Mol. Ther..

[46] Rossowska J., Anger N., Szczygiel A., Mierzejewska J., Pajtasz-Piasecka E. (2017). Intratumoral Lentivector-Mediated TGF-beta1 Gene Downregulation As a Potent Strategy for Enhancing the Antitumor Effect of Therapy Composed of Cyclophosphamide and Dendritic Cells. Front. Immunol..

[47] Anger-Gora N., Wegierek-Ciura K., Szczygiel A., Mierzejewska J., Pajtasz-Piasecka E., Rossowska J. (2021). Treatment with lentiviral vectors encoding shRNA against interleukin 10 modulates the immunosuppressive activity of murine colon carcinoma-associated myeloid-derived suppressor cells. Oncol. Lett..

[48] Suksanpaisan L., Russell S.J., Peng K.W. (2014). High scFv-receptor affinity does not enhance the antitumor activity of HER2-retargeted measles virus. Cancer Gene Ther..

[49] Friedrich K., Hanauer J.R., Prufer S., Munch R.C., Volker I., Filippis C., Jost C., Hanschmann K.M., Cattaneo R., Peng K.W. (2013). DARPin-targeting of measles virus: Unique bispecificity, effective oncolysis, and enhanced safety. Mol. Ther..

[50] Kneissl S., Zhou Q., Schwenkert M., Cosset F.L., Verhoeyen E., Buchholz C.J. (2013). CD19 and CD20 targeted vectors induce minimal activation of resting B lymphocytes. PLoS ONE.

[51] Zhou Q., Schneider I.C., Edes I., Honegger A., Bach P., Schonfeld K., Schambach A., Wels W.S., Kneissl S., Uckert W. (2012). T-cell receptor gene transfer exclusively to human CD8^+^ cells enhances tumor cell killing. Blood.

[52] Marodon G., Mouly E., Blair E.J., Frisen C., Lemoine F.M., Klatzmann D. (2003). Specific transgene expression in human and mouse CD4^+^ cells using lentiviral vectors with regulatory sequences from the CD4 gene. Blood.

[53] Nikolic J., Belot L., Raux H., Legrand P., Gaudin Y., Albertini A.A. (2018). Structural basis for the recognition of LDL-receptor family members by VSV glycoprotein. Nat. Commun..

[54] Yu B., Shi Q., Belk J.A., Yost K.E., Parker K.R., Li R., Liu B.B., Huang H., Lingwood D., Greenleaf W.J. (2022). Engineered cell entry links receptor biology with single-cell genomics. Cell.

[55] Liang M., Yan M., Lu Y., Chen I.S. (2013). Retargeting vesicular stomatitis virus glycoprotein pseudotyped lentiviral vectors with enhanced stability by in situ synthesized polymer shell. Hum. Gene Ther. Methods.

[56] Agarwal S., Hanauer J.D.S., Frank A.M., Riechert V., Thalheimer F.B., Buchholz C.J. (2020). In Vivo Generation of CAR T Cells Selectively in Human CD4^+^ Lymphocytes. Mol. Ther..

[57] Topalian S.L., Taube J.M., Pardoll D.M. (2020). Neoadjuvant checkpoint blockade for cancer immunotherapy. Science.

[58] Patsoukis N., Brown J., Petkova V., Liu F., Li L., Boussiotis V.A. (2012). Selective effects of PD-1 on Akt and Ras pathways regulate molecular components of the cell cycle and inhibit T cell proliferation. Sci. Signal..

[59] Ratnikova N.M., Kravchenko Y., Ivanova A., Zhuchkov V., Frolova E., Chumakov S. (2024). A Novel Anti-CD47 Nanobody Tetramer for Cancer Therapy. Antibodies.

[60] Moll M., Klenk H.D., Maisner A. (2002). Importance of the cytoplasmic tails of the measles virus glycoproteins for fusogenic activity and the generation of recombinant measles viruses. J. Virol..

[61] Masse N., Barrett T., Muller C.P., Wild T.F., Buckland R. (2002). Identification of a second major site for CD46 binding in the hemagglutinin protein from a laboratory strain of measles virus (MV): Potential consequences for wild-type MV infection. J. Virol..

[62] Xing X.X., Zhao X.Y., Dong Y.C. (2018). Down-regulation of Treg by interference of enhances the killing effect of CIK on leukemia cell HL-60. Eur. Rev. Med. Pharmacol. Sci..

[63] Kumagai S., Togashi Y., Kamada T., Sugiyama E., Nishinakamura H., Takeuchi Y., Vitaly K., Itahashi K., Maeda Y., Matsui S. (2020). The PD-1 expression balance between effector and regulatory T cells predicts the clinical efficacy of PD-1 blockade therapies. Nat. Immunol..

[64] Kamada T., Togashi Y., Tay C., Ha D., Sasaki A., Nakamura Y., Sato E., Fukuoka S., Tada Y., Tanaka A. (2019). PD-1^+^ regulatory T cells amplified by PD-1 blockade promote hyperprogression of cancer. Proc. Natl. Acad. Sci. USA.

[65] Otano I., Escors D., Schurich A., Singh H., Robertson F., Davidson B.R., Fusai G., Vargas F.A., Tan Z.M.D., Aw J.Y.J. (2018). Molecular Recalibration of PD-1+ Antigen-Specific T Cells from Blood and Liver. Mol. Ther..

[66] Shinohara T., Taniwaki M., Ishida Y., Kawaichi M., Honjo T. (1994). Structure and chromosomal localization of the human PD-1 gene (PDCD1). Genomics.

[67] Tarique M., Naz H., Suhail M., Turan A., Saini C., Muhammad N., Shankar H., Zughaibi T.A., Khan T.H., Khanna N. (2023). Differential expression of programmed death 1 (PD-1) on various immune cells and its role in human leprosy. Front. Immunol..

[68] Chen M., Bie L., Ying J. (2023). Cancer cell-intrinsic PD-1: Its role in malignant progression and immunotherapy. Biomed. Pharmacother..

