# Supplementary material for: PD1-Targeted Transgene Delivery to Treg Cells
Source: Viruses. 2024 Dec 19;16(12):1940. doi: 10.3390/v16121940 (PMC11680301; doi:10.3390/v16121940)
Supplement: Supplementary file 1 [file viruses-16-01940-s001.zip › viruses-3319973-supplementary.pdf]

Supplemental figures

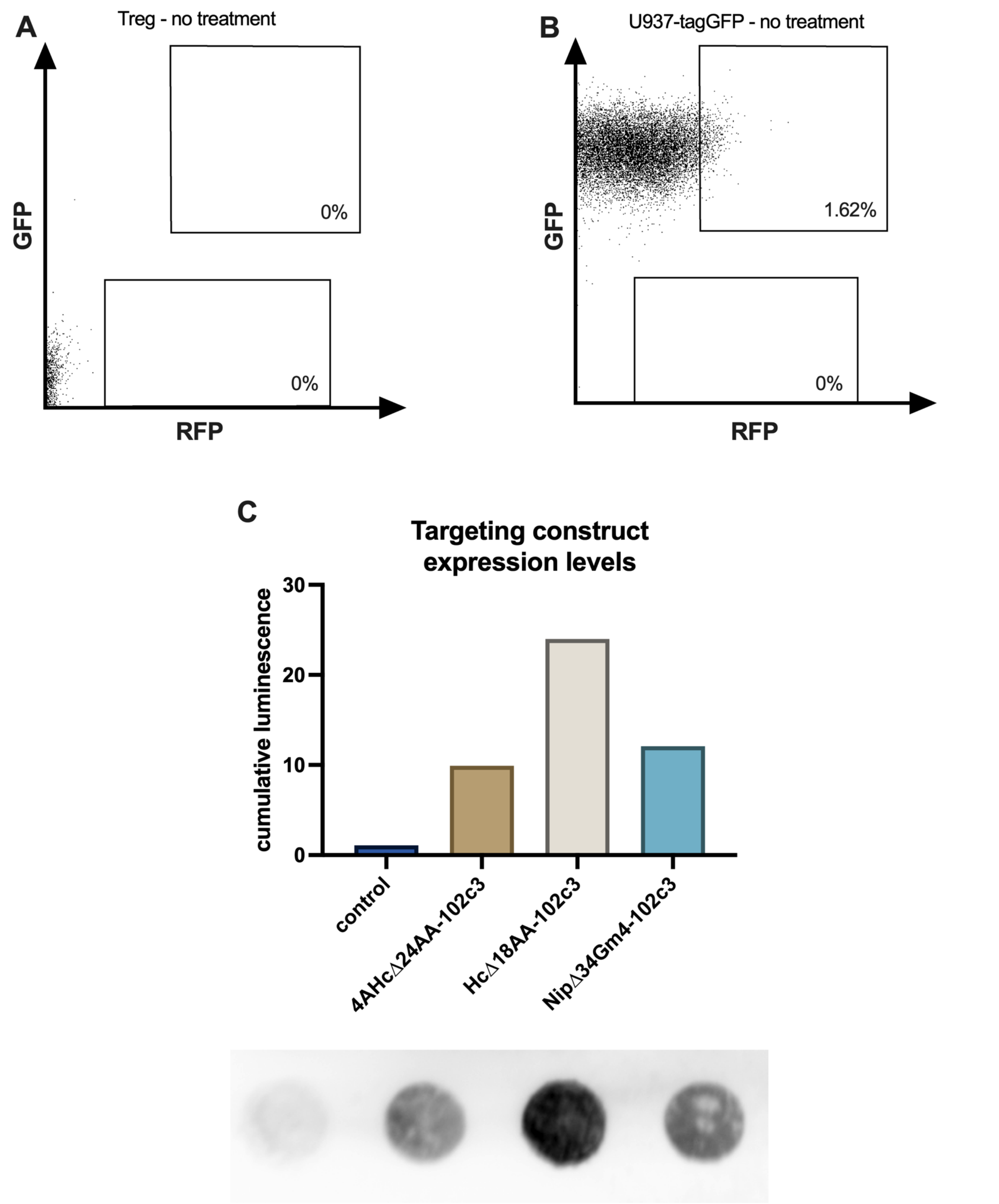

Figure S1. Control cytomentry measurements and targeting construct expression levels. Cytometry measurements of non-transduced $CD4^{+}$ $PD1^{+}$ T lymphocytes (A), and non-transduced U937-tagGFP (B). C – Dot-blot quantitation of expression levels of pCG-4AHcΔ24AA-102c3, pCG-HcΔ18AA-102c3, and pCG-NipΔ34Gm4-102c3 performed with anti-6HIS antibodies conjugated to HRP. Transfection was carried out with equal amounts of each plasmid (integral density multiplied by 10^{-9} is plotted).

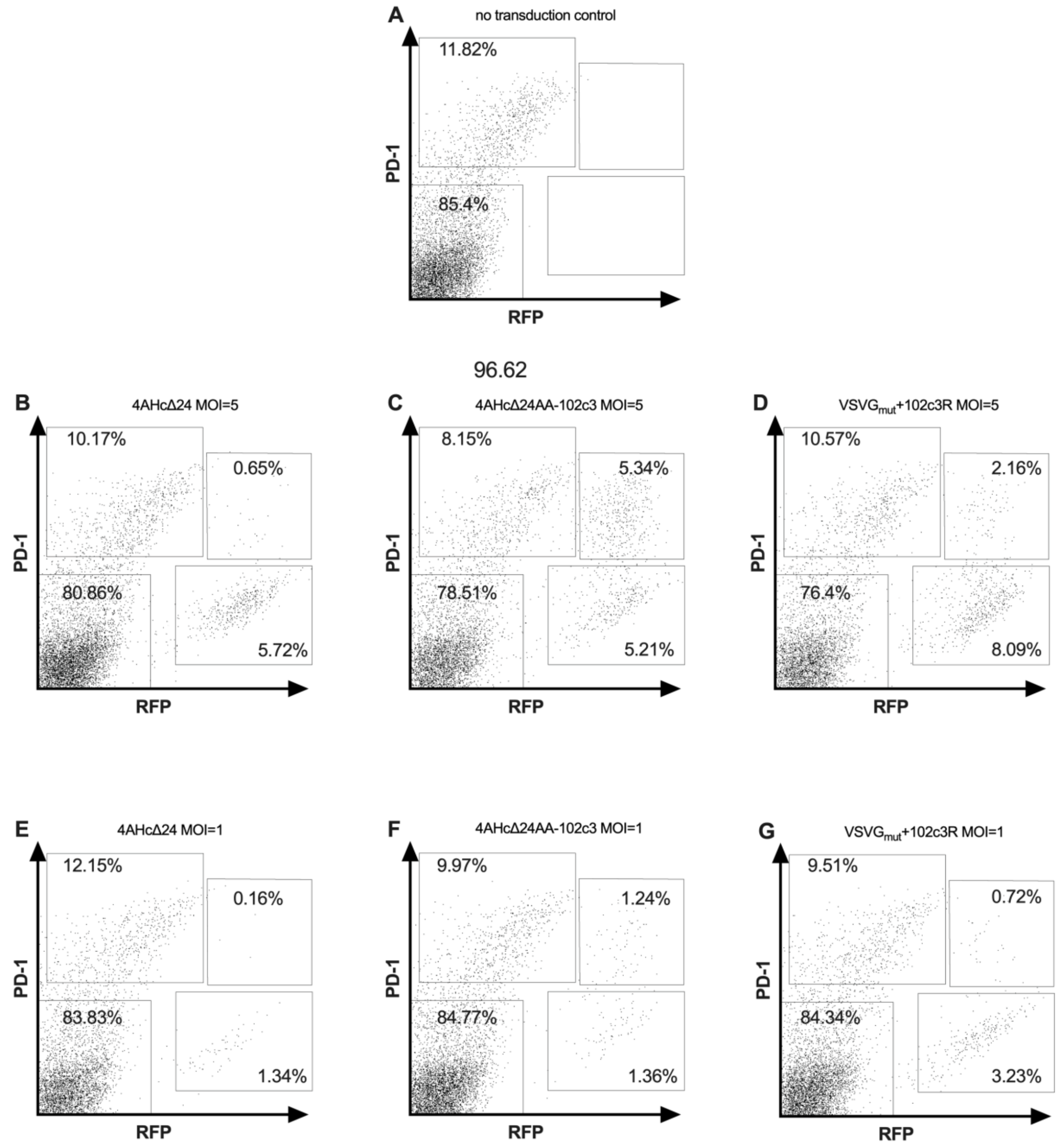

Figure S2. Cytometry analysis of $PD1^+$ $CD4^+$ T lymphocytes transduced with tagRFP-expressing pseudotyped lentivectors. Cytometry analysis of $CD4^+$ T lymphocytes briefly induced for PD1 expression (treatment 3, Fig. 2A), transduced with tagRFP-expressing lentivectors pseudotyped as follows: 4AHcΔ24 at MOI=5 (B), 4AHcΔ24AA-102c3 at MOI=5 (C), VSVGmut+102c3R at MOI=5 (D), 4AHcΔ24 at MOI=1 (E), 4AHcΔ24AA-102c3 at MOI=1 (F), VSVGmut+102c3R at MOI=1 (G), and a no-transduction control (A). Cells were stained with FITC-conjugated anti-human PD1 antibody (BioLegend).
